# Two different populations of Müller cells stabilize the structure of the fovea: an optical coherence tomography study

**DOI:** 10.1007/s10792-020-01477-3

**Published:** 2020-07-06

**Authors:** Andreas Bringmann, Jan Darius Unterlauft, Renate Wiedemann, Thomas Barth, Matus Rehak, Peter Wiedemann

**Affiliations:** Department of Ophthalmology and Eye Hospital, University of Leipzig, Liebigstrasse 10-14, 04103 Leipzig, Germany

**Keywords:** Macular telangiectasia, Vitreomacular traction, Lamellar macular hole, Full-thickness macular hole, Fovea, Müller glia

## Abstract

**Purpose:**

To document with spectral-domain optical coherence tomography the structural stabilization of the fovea and the sealing of outer macular defects by Müller cells.

**Methods:**

A retrospective case series of 45 eyes of 34 patients is described.

**Results:**

In cases of a cystic disruption of the foveola as in macular telangiectasia type 2 and vitreomacular traction, the Müller cell cone provides the structural stability of the fovea. In cases of a detachment or disruption of the Müller cell cone, e.g., in foveal pseudocysts, outer lamellar holes, and degenerative and tractional lamellar holes, Müller cells of the foveal walls may provide the structural stability of the fovea by the formation of a hyperreflective external limiting membrane (ELM) which bridges the holes in the central outer nuclear layer (ONL). Müller cells of the foveal walls and parafovea mediate the regeneration of the foveal architecture in cases of outer lamellar and full-thickness macular holes. The regeneration proceeds by a centripetal displacement of photoreceptor cell somata which closes the holes in the central ONL. The closure may be supported by the formation of a glial tissue band at the ELM which seals the hole.

**Conclusions:**

The Müller cell cone provides the foveal stability in cases of a cystic disruption of the foveola. The structural stability of the outer foveal layers is mainly provided by the Müller cells of the foveal walls and parafovea; these cells also mediate the regeneration of the outer fovea in cases of a defect of the central ONL.

## Introduction

The structural stability of the fovea is provided by two populations of Müller glial cells: specialized cells which form the so-called Müller cell cone in the foveola, and Müller cells of the foveal walls and parafovea which have a characteristic *z*-shape because their outer processes run horizontally or obliquely through the Henle fiber layer (HFL) toward the foveal center [1–4]. The Müller cell cone may also contribute to the maintenance of the integrity of the foveal walls which surround the central foveola. A detachment of the horizontal layer of the Müller cell cone from the HFL/outer nuclear layer (ONL) in the foveola by anteroposterior traction exerted by the partially detached posterior hyaloid and the disruption of the vertical stalk of the Müller cell cone is often associated with an elevation of the inner layers of the foveal walls (nerve fiber layer [NFL] to the outer plexiform layer [OPL]); this may produce cystic cavities between the OPL and HFL, and within the inner nuclear layer (INL). Elevation of the inner layers of the foveal walls was shown to be implicated in the development of foveal pseudocysts, lamellar macular defects, and full-thickness macular holes (FTMH) [5–12].

The structural stability of the outer layers of the fovea (HFL, ONL) is suggested to be mainly provided by the outer processes of the Müller cells of the foveal walls and parafovea [4]. These processes surround the fibers and somata of photoreceptor cells, and constitute, together with the photoreceptor cells, the external limiting membrane (ELM) [3, 13]. Photoreceptor and Müller cells are tightly glued together in the HFL and ONL, and at the ELM [14, 15]. At the ELM, Müller cells contain contractile rings of filamentous actin which enclose the photoreceptors; these rings are associated with the junctions between Müller and photoreceptor cells and form a structural meshwork in which the photoreceptors are embedded [16]. The spatial arrangement of the Müller cell processes in the central ONL and the morphology of lamellar holes without disruption of the outer retinal layers may suggest that Müller cells of the foveal walls stabilize the architecture of the ONL, especially near and at the ELM. The stalk of the Müller cell cone does not contribute to the formation of the ELM because the cells of the Müller cell cone have no direct contact to photoreceptor cells [4, 13]; this may ensure that the central ELM remains intact after a disruption of the stalk of the Müller cell cone.

Müller cells of the foveal walls and parafovea are also implicated in the regeneration of the outer retinal structure after surgical or spontaneous closure of FTMH [17–19]. Following the drop of the elevated inner layers of the foveal walls, which allows a reattachment of the outer retina to the retinal pigment epithelium (RPE), the remaining gaps in the central ONL and ELM are closed by a centripetal displacement of the photoreceptor cell somata likely mediated by a concentric contraction of the outer processes of the Müller cells of the foveal walls [18]. It is unclear whether this Müller cell-mediated regeneration of the outer retina is also observed in other types of outer retinal defects, e.g., outer lamellar holes [7]. It was recently described that the spontaneous closure of an outer lamellar hole in a patient with macular telangiectasia type 2 started as bridging at the level of the ELM and the outer part of the ONL; the authors suggested that extension or proliferation of Müller cells is the main mechanism for this phenomenon [20]. Here, we describe that one mode implicated in the structural stabilization of the outer fovea after disruption of the central ONL and ELM is the formation of a hyperreflective glial tissue band at the ELM which bridges holes in the outer retina. Cases of a tractional disruption of the Müller cell cone, foveal (pseudo)cysts, degenerative, tractional, and outer lamellar holes, and FTMH are included in the study. A FTMH develops by a disruption of both the Müller cell cone and the ELM in the foveola. An outer lamellar hole is characterized by a detachment of the inner Müller cell layer from the HFL/ONL and a large pseudocyst in the foveola which is associated with a disruption of the ELM and a dehiscence of the central ONL [7]. An outer lamellar hole often represents an early stage of FTMH formation [7, 12, 21]. In addition, we describe various cases of macular telangiectasia type 2 which are characterized by a cystic degeneration of the foveola due to the paracentral loss of photoreceptor cells [22, 23]; in these cases, the shape of the central fovea is maintained by the Müller cell cone because no tractions are present which detach or tear the cone.

## Methods

This retrospective study followed the ethical standards of the Declaration of Helsinki for research involving human subjects. The patients were referred to the Department of Ophthalmology, University of Leipzig, Germany, between February 2008 and September 2019. Thirty one patients with different types of partial-thickness macular defects and three patients with surgical closure of a FTMH were included in the study. Cross-sectional images of the macular area were obtained with spectral-domain optical coherence tomography (SD-OCT; Spectralis, Heidelberg Engineering, Heidelberg, Germany). Best-corrected visual acuity (BCVA) was determined with a Snellen chart and is given in decimal units.

**Fig. 1:**
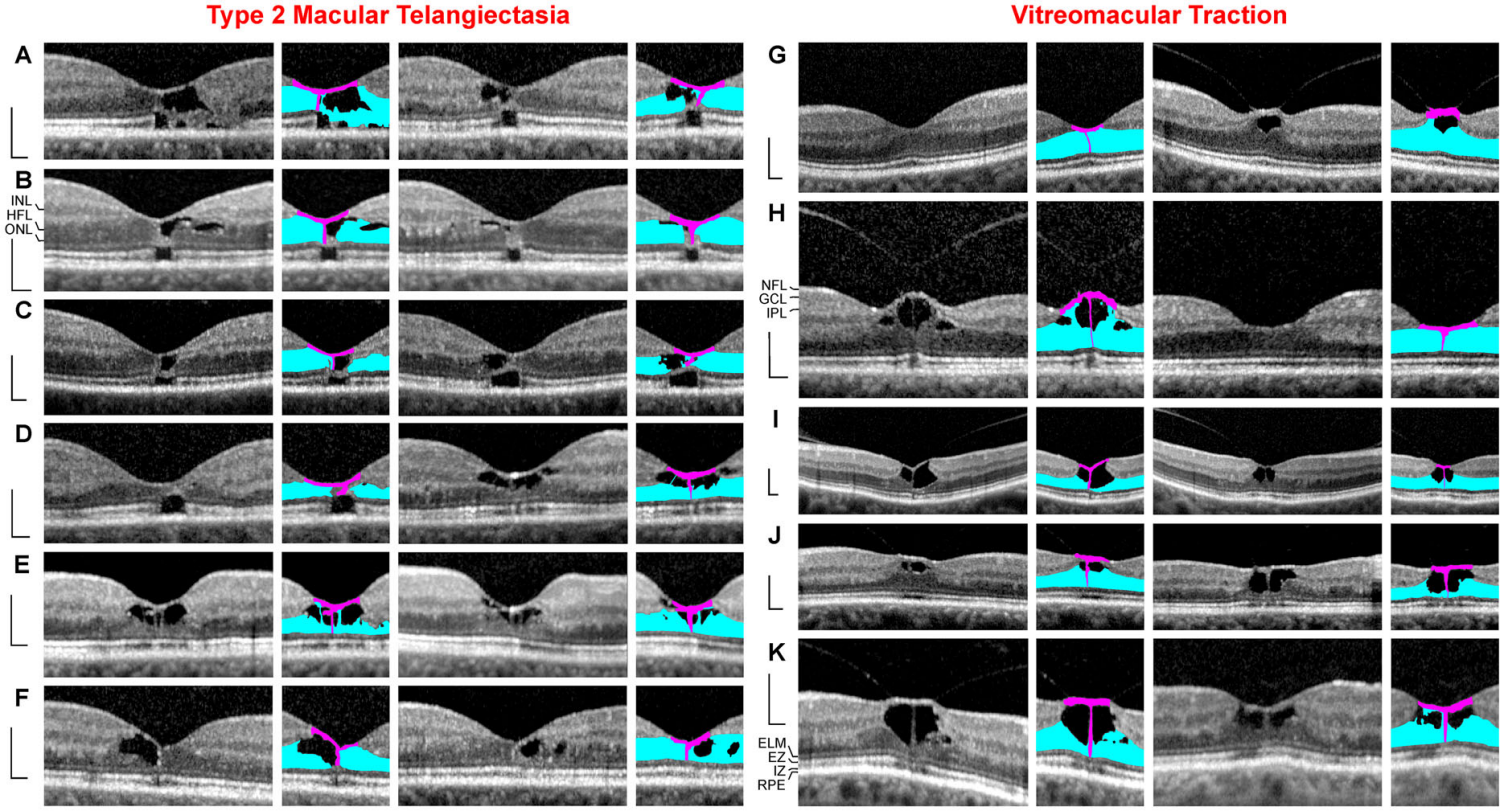
Müller cell cone provides the stability of the foveal center in cases of cystic disruption of the foveola. **a**–**f** Macular telangiectasia type 2. The images show linear scans through the fovea and parafovea in the left (left side) and right eyes (right side) of 6 patients. **g**–**k** Vitreomacular traction. The images show linear scans through the fovea and parafovea in the left (left side) and right eyes (right side) of 5 patients. The *smaller images* beside the scans show the Müller cell cone in pink and the Henle fiber (HFL)/outer nuclear layers (ONL) of the fovea in blue. Scale bars, 200 µm. ELM, external limiting membrane; EZ, ellipsoid zone; GCL, ganglion cell layer; INL, inner nuclear layer; IPL, inner plexiform layer; IZ, interdigitation zone; NFL, nerve fiber layer; OPL, outer plexiform layer; RPE, retinal pigment epithelium

**Fig. 2:**
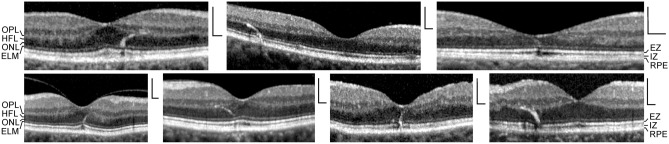
Curved hyperreflective structures in the Henle fiber layer (HFL) and outer nuclear layer (ONL), which are associated with small sites of lost photoreceptor integrity, likely reflect gliosis of the outer processes of the Müller cells of the foveal walls and parafovea. The images show SD-OCT scans through the foveas of 7 subjects. Scale bars, 200 µm. ELM, external limiting membrane; EZ, ellipsoid zone; IZ, interdigitation zone; OPL, outer plexiform layer; RPE, retinal pigment epithelium

**Fig. 3:**
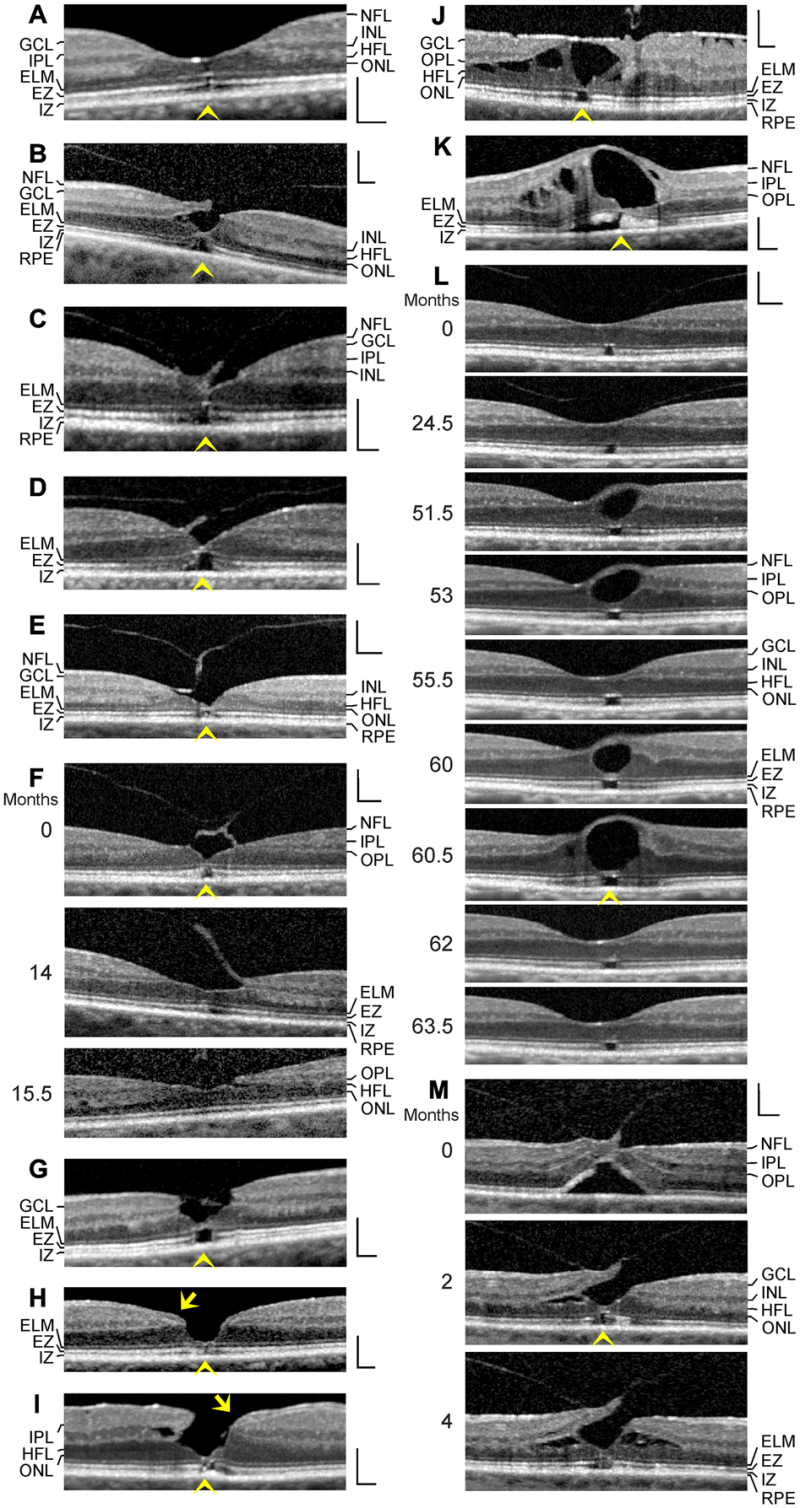
Stability of the outer foveal structure may be supported by a hyperreflective glial tissue band at the external limiting membrane (ELM) (arrowheads) in different types of outer macular defects. The images show scans through the fovea and parafovea in eyes of different patients. **a** This fovea had a gap in the central outer nuclear layer (ONL) and a hyperreflective band which sealed the gap at the ELM. **b**–**e** Four cases of a tractional disruption of the Müller cell cone in the foveola. **f** Foveal regeneration after a tractional detachment and disruption of the Müller cell cone in the foveola. The months after the first visit (0) are indicated left of the images. Pars plana vitrectomy with internal limiting membrane peeling was performed 1.3 months after the first visit. **g** Another case of a tractional disruption of the Müller cell cone. **h** Impending degenerative lamellar hole. The arrow indicates lamellar macular hole-associated epiretinal proliferation (LHEP). **i** Degenerative lamellar hole. The arrow indicates LHEP. **j** Foveal pseudocyst. Note the presence of both epiretinal membranes and vitreofoveal adhesion. **k** Tissue disruption by a foveal cyst. **l** Multiple development and resolution of a cyst in the fovea. **m** Development of a tractional lamellar hole by anteroposterior traction exerted by the partially detached posterior hyaloid. Scale bars, 200 µm. EZ, ellipsoid zone; GCL, ganglion cell layer; HFL, Henle fiber layer; INL, inner nuclear layer; IPL, inner plexiform layer; IZ, interdigitation zone; NFL, nerve fiber layer; OPL, outer plexiform layer; RPE, retinal pigment epithelium

All patients were Caucasians. SD-OCT images of the macular regions of both eyes of 6 patients with macular telangiectasia type 2 were investigated (Fig. 1a–f; 4 women, 2 men; mean ± S.D. age, 59.7 ± 6.7 years; range 49–68 years). The mean BCVA was 0.73 ± 0.21 (range, 0.4–1.0). SD-OCT scans of 5 further women showed vitreomacular traction without disruption of the Müller cell cone in one or both eyes (Fig. 1g–k; mean age, 68.6 ± 14.3 years; range, 45–83 years; mean BCVA of the affected eyes, 0.55 ± 0.21, range 0.3–1.0). SD-OCT scans through the fovea of 7 subjects (mean age, 65.6 ± 14.7 years; range, 48–81 years) showed curved hyperreflective structures in the outer retina (Fig. 2). In addition, 13 patients with different types of tractionally induced macular defects in one eye were investigated (Fig. 3a–m; 9 women, 4 men; mean age, 67.4 ± 17.7 years, range 16–86 years). The BCVA of the eyes with macular defects ranged from 0.3 to 0.9 (mean, 0.60 ± 0.17).

**Fig. 4:**
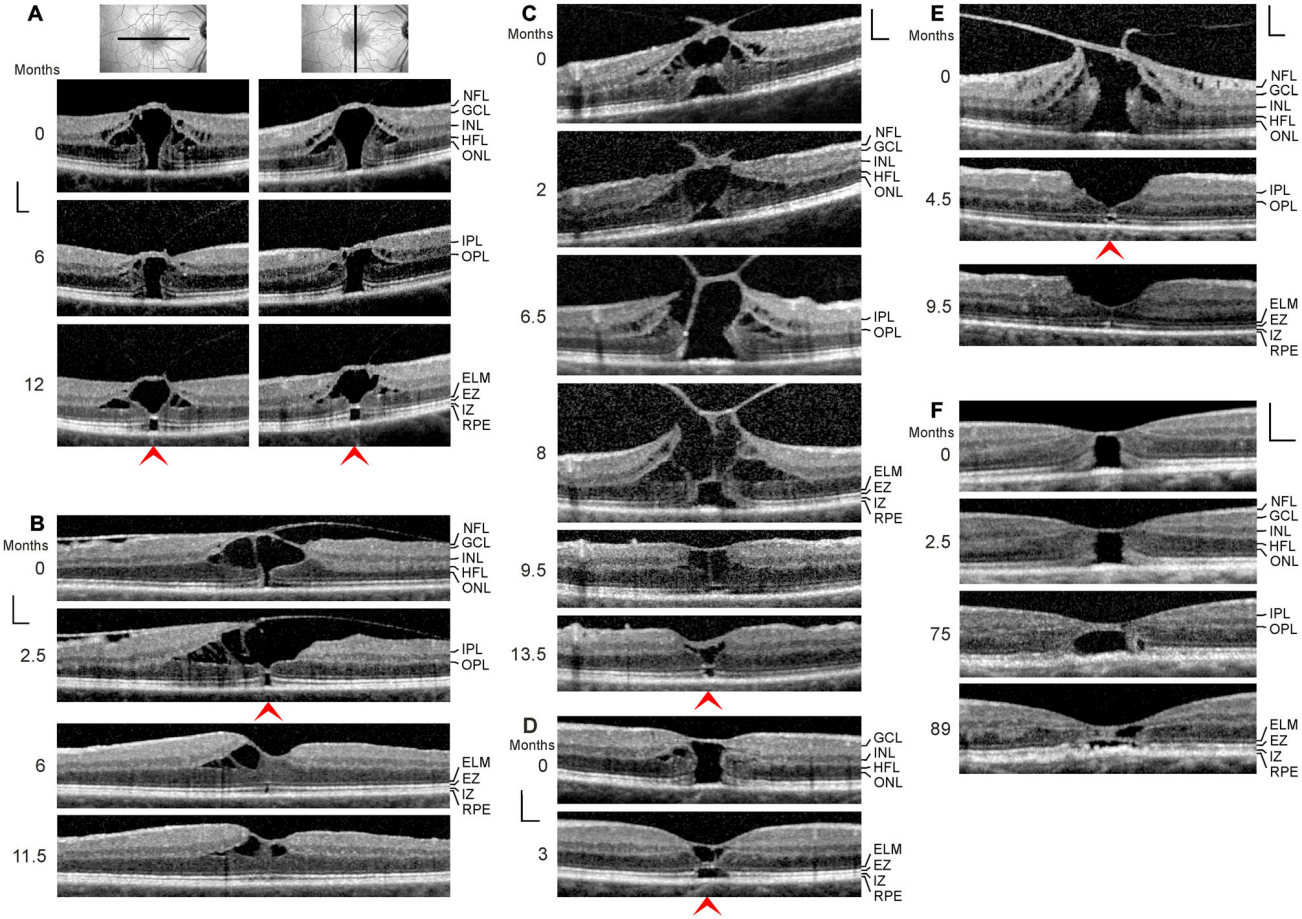
Regeneration of outer lamellar holes involves the formation of a hyperreflective glial tissue band which bridges the hole in the outer retina at the external limiting membrane (ELM) and the outer part of the outer nuclear layer (ONL) (arrowheads). The months after the first visit (0) are indicated left of the images. **a** A case of an outer lamellar hole. The orientations of the scans are shown above. **b** An outer lamellar hole which evolved into a tractional lamellar hole within 2.5 months after the first examination. Vitrectomy with internal limiting membrane (ILM) peeling was performed 2.6 months after the first visit. **c** Tractional generation and partial closure of an outer lamellar hole. Vitrectomy with ILM peeling was performed 8.1 months after the first visit. **d** Another outer lamellar hole. **e** Tractional generation and closure of an outer lamellar hole. Vitrectomy with ILM peeling was performed 0.5 month after the first visit. **f** Partial restoration of the contour of the central fovea after regeneration of the central ELM in an outer lamellar hole. Scale bars, 200 µm. EZ, ellipsoid zone; GCL, ganglion cell layer; HFL, Henle fiber layer; INL, inner nuclear layer; IPL, inner plexiform layer; IZ, interdigitation zone; NFL, nerve fiber layer; OPL, outer plexiform layer; RPE, retinal pigment epithelium

**Fig. 5:**
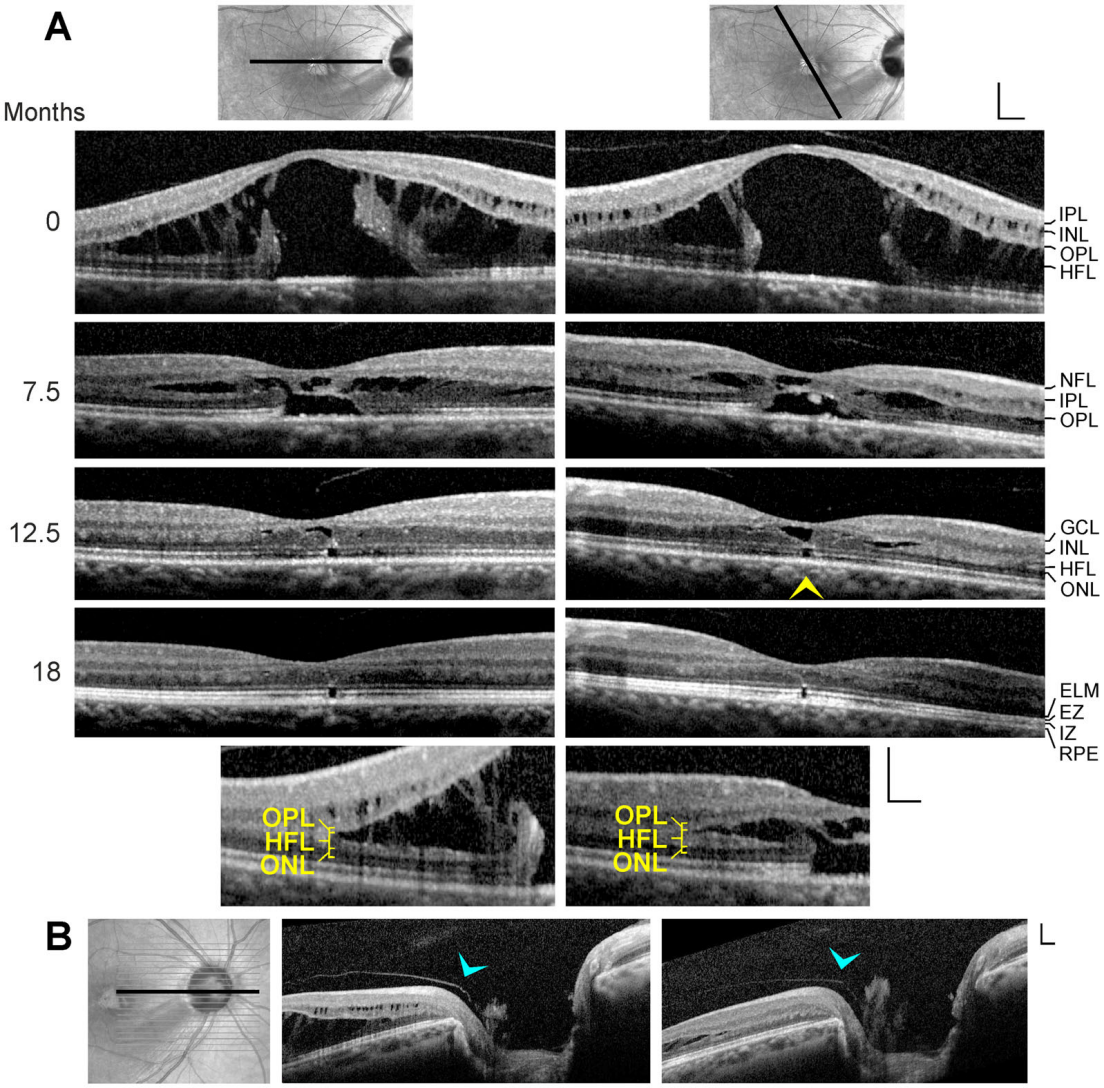
Regeneration of the foveal morphology after spontaneous reattachment of the fovea in a case of serous macular detachment associated with a congenital optic pit. The images show linear scans through the fovea and parafovea (**a**) and the optic disk (**b**) of the right eye of a 30-year-old woman. **a** The months after the first visit (0) are indicated left of the images. The orientations of the scans are shown above. The yellow arrowhead indicates the hyperreflective glial tissue band which bridged the cyst in the foveola at the external limiting membrane (ELM) and the outer part of the outer nuclear layer (ONL). The smaller images below show scans through the fovea and parafovea obtained at (left) and 7.5 months after the first visit (right) at higher magnification. Note that the tissue split between the outer plexiform layer (OPL) and Henle fiber layer (HFL). **b** The scans were recorded during (left) and 7.5 months after the first visit (right). The blue arrowheads indicate vitreal adhesion at the prepapillary glial material. Scale bars, 200 µm. EZ, ellipsoid zone; GCL, ganglion cell layer; INL, inner nuclear layer; IPL, inner plexiform layer; IZ, interdigitation zone; NFL, nerve fiber layer; RPE, retinal pigment epithelium

SD-OCT images recorded in one eye of 6 patients revealed the presence of an outer lamellar hole (Fig. 4a–f; 5 women, 1 man; mean age, 65.6 ± 6.3 years, range 55–72 years). The mean BCVA of these eyes was 0.38 ± 0.17 (range, 0.16–0.6). A 30-year-old woman had central visual disturbance and metamorphopsia in the right eye (BCVA, 0.16); SD-OCT showed serous macular detachment associated with a congenital optic disk pit (Fig. 5a, b). The foveal morphology regenerated spontaneously during the follow up, and BCVA improved to 0.6.

**Fig. 6:**
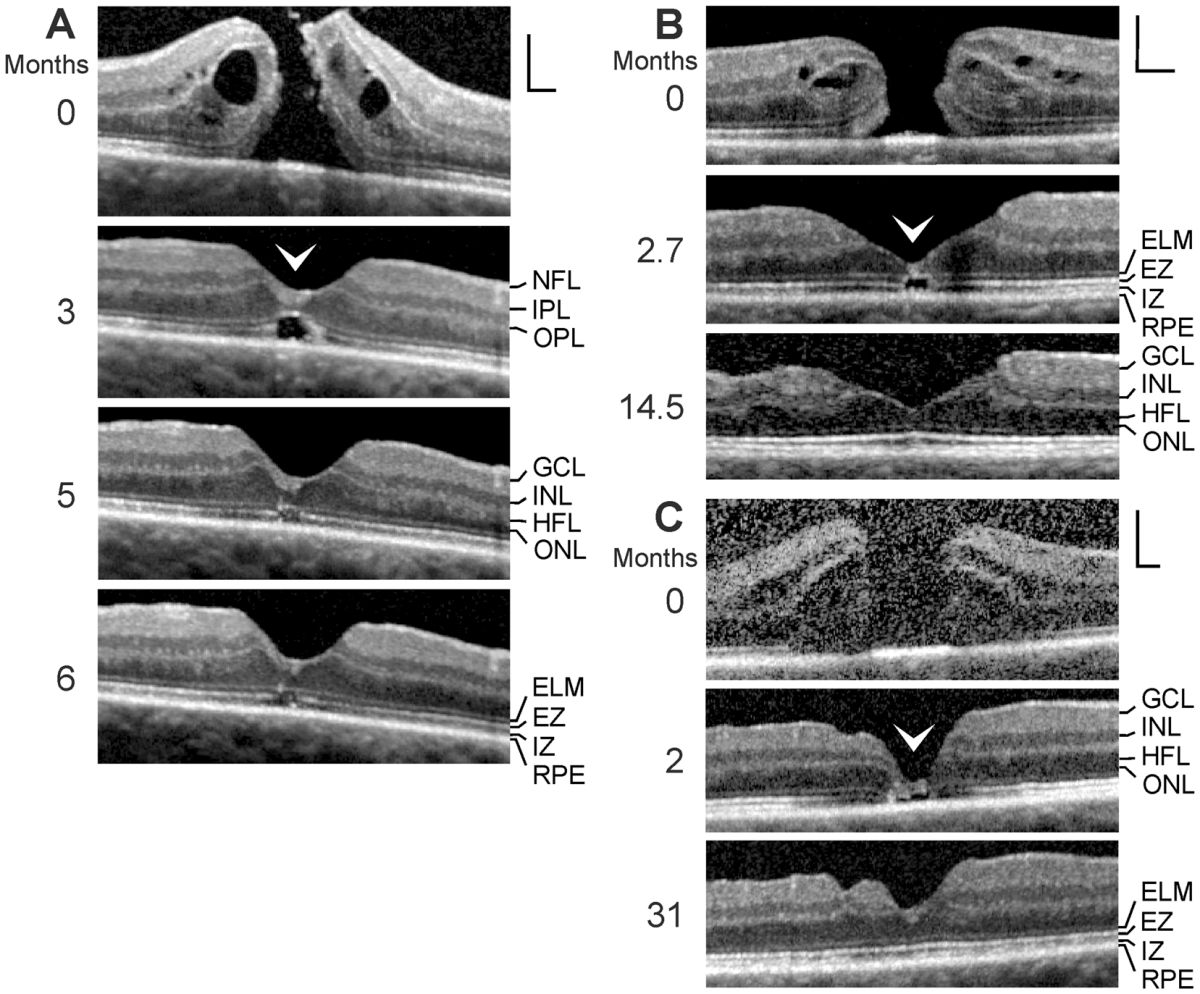
Regeneration of the foveal shape after closure of a full-thickness macular hole (FTMH) may involve the formation of a glial tissue with medium reflectivity which seals the hole at the outer layers (arrowheads). The months after the first examination (0) are indicated left of the images. **a**–**c** Three cases of surgical closure of a FTMH. Vitrectomy with internal limiting membrane peeling was performed one (**a**), 1.5 (**b**), and 0.5 months (**c**) after the first visit, respectively. Scale bars, 200 µm. ELM, external limiting membrane; EZ, ellipsoid zone; GCL, ganglion cell layer; HFL, Henle fiber layer; INL, inner nuclear layer; IPL, inner plexiform layer; IZ, interdigitation zone; NFL, nerve fiber layer; ONL, outer nuclear layer; OPL, outer plexiform layer; RPE, retinal pigment epithelium

The foveal regeneration after surgical closure of a FTMH with standard pars plana vitrectomy, internal limiting membrane (ILM) peeling, and SF6 tamponade in one eye of 3 women was also investigated (Fig. 6a–c; mean age, 71.0 ± 7.8 years, range 62–76 years). The mean BCVA of these eyes before surgery was 0.27 ± 0.03 (range, 0.25–0.30).

## Results

### Macular telangiectasia type 2

Macular telangiectasia type 2 is characterized by a progressive bilateral cystic degeneration of the ONL in the fovea because of a loss of photoreceptor cells [22, 23]. Because there are no tractional forces which disrupt the tissue integrity, the foveal contour remains normal; the shape of the foveal pit is maintained by the nondisrupted Müller cell cone which consists of the innermost horizontal layer of the foveola and the vertical stalk in the center of the foveola [4]. This is illustrated by the cases of macular telangiectasia type 2 in both eyes of 6 patients (Fig. 1a–f). Cystic cavities in the foveola mainly developed between the horizontal layer of the Müller cell cone and the HFL/ONL; the vertical stalk of the Müller cell cone prevented an elevation of the horizontal layer and thus maintained the shape of the foveal pit. The degeneration of photoreceptor cells was associated with a loss of the photoreceptor integrity, as indicated by the defects of the ellipsoid zone (EZ) and interdigitation zone (IZ) lines (Fig. 1a–c). In areas of a full degeneration of the photoreceptors, e.g., in the cases shown in Fig. 1b, c, glial tissues near and at the ELM formed by the outer processes of the Müller cells of the foveal walls contributed to the stabilization of the central foveal shape.

### Vitreomacular traction without disruption of the Müller cell cone

The Müller cell cone also maintains the foveal integrity in cases of vitreofoveal traction; however, in these cases, the foveal contour is deteriorated. SD-OCT scans of various eyes of 5 patients showed vitreomacular traction without a disruption of the Müller cell cone (Fig. 1g–k). This was associated with the formation of foveal pseudocysts due to a detachment of the horizontal layer of the Müller cell cone from the HFL/ONL in the foveola and an elongation or disruption of the vertical stalk of the cone. Foveal pseudocysts were also associated with an elevation of the inner layers of the foveal walls (NFL to OPL) and, in some cases, small cystic cavities between the OPL and HFL (Fig. 1h, k). In the fovea shown in Fig. 1I (left side), the central outer retina is stabilized only by a thin hyperreflective glial tissue near the ELM.

Figure 3a shows a fovea with a dehiscence of the central ONL (gap diameter, 40 µm) associated with defects of the EZ and IZ lines, indicating a loss of the photoreceptor integrity. The inner Müller cell layer of the foveola and the ELM were not disrupted. The ELM which bridged the gap in the ONL was hyperreflective.

### Spatial arrangement of the outer processes of the Müller cells of the foveal walls

A small focal loss of the photoreceptor integrity, as indicated by altered reflectivities of the EZ and IZ lines, may be associated with a curved hyperreflective structure which extends between the OPL and ELM (Fig. 2). The positions of the hyperreflective structures reflect the spatial arrangement of the photoreceptor cell fibers in the HFL and ONL. It could be that the hyperreflective structures are caused by a gliosis of the outer processes of the Müller cells of the foveal walls and parafovea induced by the local degeneration of the photoreceptor cells. These processes draw centripetally from the OPL toward the ELM, surround the photoreceptor cell axons in the HFL and the photoreceptor cell somata and outer processes in the ONL, and contribute to the formation of the ELM [13, 16].

### Tractional disruption of the Müller cell cone

In cases of a tractional disruption of the Müller cell cone, Müller cells of the foveal walls and parafovea may provide the structural stability of the foveal center. Figure 3b shows a fovea in which vitreofoveal traction caused a disruption of the connection between the inner Müller cell layer of the foveola and the temporal foveal wall. This was associated with a detachment of the inner Müller cell layer from the HFL/ONL in the foveola and a dehiscence of the central outer retina; the hole in the outer retina was sealed by the ELM and a tissue of medium reflectivity, likely composed of Müller cell processes. This sealing prevented the formation of a FTMH.

Figure 3c shows another case of a tractional disruption of the Müller cell cone which was associated with a dehiscence of the central ONL; the gap in the ONL was filled by a hyperreflective tissue, likely representing the gliotic stalk of the Müller cell cone. The ELM which bridged the photoreceptor-free area in the central foveola was hyperreflective.

Figure 3d–g shows further cases of a detachment and disruption of the Müller cell cone in the foveola produced by traction exerted by the partially detached posterior hyaloid. In the cases shown in Fig. 3d, e, the detachment of the disrupted Müller cell cone resulted in a dehiscence of the central ONL. The foveal center was only stabilized by the ELM. The central borders of the ONL showed a curved shape, similar to the curved hyperreflective structures in the outer retina found in SD-OCT scans of foveas with small sites of lost photoreceptor integrity (Fig. 2). This suggests that the shape of the central ONL was stabilized by the outer processes of the Müller cells of the foveal walls, despite the dehiscence of this layer.

A similar morphology of the central fovea was observed in the case shown in Fig. 3f. Vitrectomy with ILM peeling was performed 1.3 months after the first visit. Despite the persistent detachment of the inner layer of the foveola, the thickness of the central ONL increased within 14 months after the first visit, likely by a centripetal displacement of photoreceptor cell somata; this was associated with a closure of the central defect of the EZ line. Another case of a disruption of the Müller cell cone and a dehiscence of the central ONL is shown in Fig. 3g; the photoreceptor-free area in the foveal center was bridged by the ELM.

### Degenerative lamellar holes

A disruption of the Müller cell cone may also initiate the development of a degenerative lamellar hole. Many cases of degenerative lamellar holes show the presence of an atypical proliferative tissue at the NFL termed lamellar hole-associated epiretinal proliferation (LHEP) [24–28]. Figure 3h shows a case of a disruption of the Müller cell cone which resulted in a large dehiscence of the ONL in the foveola; the foveal center kept together by the ELM. Defects of the EZ and IZ lines indicate that the center of the foveola was free of photoreceptors. This and the presence of LHEP at the vitreal side of the foveal walls may suggest that this case represents an impending degenerative lamellar hole. Figure 3i shows a case of a degenerative lamellar hole characterized by a dehiscence of the central ONL and the presence of LHEP; the photoreceptor-free area in the foveal center was bridged by a glial tissue at the level of the ELM and the outer part of the ONL.

### Foveal (pseudo)cysts

Figure 3j shows a foveal pseudocyst which was likely produced by both contractile epiretinal membranes and vitreofoveal traction. The inner layer of the foveola was detached from the HFL/ONL and elevated; apparently, a hyperreflective epiretinal membrane laid over this layer. The elevation of the inner layer of the foveola produced an elevation of the inner layers of the foveal walls (NFL to OPL) and a schistic disruption of the tissue between the OPL and HFL. Thick bundles of Henle fibers traversed the schistic cavities; the position of the bundles suggests that the elevation of the inner layers of the foveal walls caused a stretching of Müller cells which exerted an oblique traction onto the outer retina resulting in a dehiscence of the ONL in the foveola, as previously suggested for the enlargement of FTMH [11]. The gap in the central ONL was photoreceptor-free and bridged by a hyperreflective ELM.

Figure 3k shows a large edematous cyst in the fovea which was caused by branch retinal vein occlusion. The cyst produced an elevation of the inner layer of the foveola which was associated with an elevation of the inner layers of the foveal walls (NFL to IPL). The latter caused the cysts in the INL and a detachment of the central outer retina from the RPE. The large central cyst also produced a dehiscence of the ONL in the foveola; the gap in the ONL was bridged by the ELM.

Figure 3l shows a case of a twofold development and resolution of a cyst in the fovea. The scan recorded at the first visit shows vitreofoveal adhesion and an area in the foveola which was photoreceptor-free, as indicated by the EZ line defect. The ELM in the photoreceptor-free area was hyperreflective. Both episodes of foveal cyst formation were associated with a dehiscence of the central ONL. The gap in the central ONL was bridged by a hyperreflective band at the ELM and the outer part of the ONL. Thereafter, the gap in the central ONL completely closed, likely by a centripetal displacement of photoreceptor cell somata.

### Tractional lamellar hole

Figure 3m shows a case of a development of a tractional lamellar hole. The hole was produced by anteroposterior traction exerted by the partially detached posterior hyaloid. The traction caused a detachment and elevation of the central fovea from the RPE. Within 2 months after the first visit, the connection between the inner layer of the foveola and a foveal wall was disrupted; this allowed the drop of this wall while the inner layers of the opposite wall remained elevated. The reattachment of the central outer layers at the RPE was associated with a schistic splitting between the OPL and HFL of the foveal walls. The tractional forces also produced a dehiscence of the central ONL; the gap in the central ONL was sealed by the hyperreflective ELM and a tissue band of medium reflectivity, likely composed of Müller cell processes. The gap in the central ONL closed between 2 and 4 months, likely by a centripetal displacement of photoreceptor cell somata.

### Outer lamellar holes

Outer lamellar holes are characterized by a large pseudocyst in the foveola which causes a detachment of the inner foveolar layer. The elevation of the inner layer is associated with an elevation of the inner layers of the foveal walls (NFL to OPL) which causes an oblique stretching and straightening of the Müller cells of the foveal walls that transmit the tension to the outer retina. This produces a centrifugal displacement of the central ONL and photoreceptors resulting in a disruption of the ELM and a hole in the outer retina [7, 11].

Figure 4a shows an outer lamellar hole which was produced by anteroposterior traction exerted by the partially detached posterior hyaloid. Between 6 and 12 months after the first examination, the diameter of the hole in the outer retina decreased from 270 to 160 µm. This was associated with the formation of a hyperreflective tissue band which bridged the hole at the ELM and the outer part of the ONL. Figure 4b shows a small outer lamellar hole (diameter, 75 µm) which was formed by traction exerted by epiretinal membranes. Within 2.5 months after the first visit, a tractional lamellar hole was formed by the disruption of the inner layer of the foveola and the formation of a hyperreflective glial tissue band which sealed the hole in the outer retina at the ELM and the outer part of the ONL. After vitrectomy with ILM peeling performed 2.6 months after the first examination, the foveal morphology regenerated partially. The hole in the central ONL was closed after 6 months, and the defects of the EZ and IZ lines were closed 11.5 months. The ELM in front of the EZ and IZ lines defects was hyperreflective (6 months). The hyperreflectivity disappeared with the closure of the EZ and IZ line defects (11.5 months).

The SD-OCT scans in Fig. 4c show another case of a tractional development and partial closure of an outer lamellar hole. Between 6.5 and 8 months after the first visit, the central ELM regenerated. Pars plana vitrectomy with ILM peeling performed 8.1 months after the first visit removed the vitreofoveal traction which resulted in a drop of the elevated foveal walls associated with a reattachment of the central photoreceptors at the RPE. The diameter of the hole in the outer retina decreased from 270 to 120 µm between 8 and 9.5 months. The ELM which bridged the hole in the outer retina was hyperreflective. The foveal shape regenerated further between 9.5 and 13.5 months. The diameter of the outer retinal hole decreased to 110 µm, and the hole was bridged by a hyperreflective band at the ELM and the outer part of the ONL. In the case of an outer lamellar hole shown in Fig. 4d, the cystic cavities in the foveal walls resolved within 3 months after the first visit; this was associated with a drop of the elevated inner layers of the foveal walls and the central ONL, and a decrease in the diameter of the hole in the outer retina from 290 to 200 µm. The hole in the outer retina was sealed by a hyperreflective tissue band at the ELM and the outer part of the ONL.

The SD-OCT scans in Fig. 4e show a further example of a tractional generation and surgical closure of an outer lamellar hole. The removal of the vitreofoveal traction by vitrectomy with ILM peeling performed 0.5 month after the first visit resulted in a resolution of the cystic cavities in the foveal walls which allowed a drop of the elevated inner layers of the walls and a reattachment of the central outer retina at the RPE (4.5 months). The hole in the central outer retina was sealed by a hyperreflective ELM and a tissue of medium reflectivity, likely composed of Müller cell processes. Between 4.5 and 9.5 months, the central ONL thickened, likely by a centripetal displacement of photorecptor cell somata; this was associated with a decrease in the diameter of the central photoreceptor-free area and a disappearance of the hyperreflectivity of the ELM.

Figure 4f shows an outer lamellar hole without cystic cavities in the foveal walls. There was a dehiscence of the central ONL associated with defects of the ELM, EZ, and IZ lines. Between 2.5 and 75 months after the first examination, the ELM in the foveal center regenerated; the space between the inner layer of the Müller cell cone and the ELM was filled by a tissue of medium reflectivity, likely composed of Müller cells. The central ONL was not displaced toward the foveal center. In the further course, the distance between the central ELM and the RPE decreased (perhaps by a horizontal contraction of the ELM); this was associated with a partial restoration of the foveal shape, i.e., a thickening of the foveal center and a deepening of the foveal pit. However, the foveal center did not contain an ONL and photoreceptors and was composed of cystic spaces and a glial tissue of medium reflectivity.

### Serous macular detachment

Figure 5b shows a case of a congenital optic pit associated with serous macular detachment (Fig. 5a). The foveal shape resembled that of a large outer lamellar hole with a high elevation of the inner layers of the foveal walls (NFL to OPL) which produced a schisis between the OPL and HFL; the detached, nondisrupted inner layer of the foveola kept the elevated foveal walls together. Bundles of Henle fibers traversed the schistic cavities between the OPL and HFL. The high elevation of the foveal walls also caused a disruption and elevation of the central outer retina; the ONL and photoreceptors were detached from the RPE and centrifugally displaced toward the foveal walls which produced a broad horizontal gap in the ONL (diameter, 850 µm) and wide defects of the central ELM, EZ, and IZ lines. The detachment of the central ONL apparently resulted from an oblique anterior traction exerted by the Müller cells of the foveal walls onto the central photoreceptor cells [11].

During the next 18 months, the foveal morphology regenerated spontaneously (Fig. 5a). Within 7.5 months, the elevated central fovea dropped down; this was associated with a considerable decrease in the size of the schistic cavities and a reattachment of the ONL and photoreceptors at the RPE in the foveal walls. However, the central fovea contained cystic spaces without an ONL and photoreceptors; there was a relatively wide area which was photoreceptor-free (diameter, 540 µm). The hyperreflective external layer of the foveola represents the regenerated ELM. Between 7.5 and 12.5 months, the gap in the central ONL closed by a centripetal displacement of the ONL of the foveal walls; this was associated with a decrease in the distance between the central hyperreflective ELM and the RPE. Between 12.5 and 18 months, the gap in the central ONL fully closed. There remained a small photoreceptor-free area which was associated with a hyperreflective ELM and a curved hyperreflectivity in the ONL. The latter may indicate that the outer processes of the Müller cells of the foveal walls were gliotic in this area.

### Foveal regeneration after surgical closure of a FTMH

The regeneration of the foveal architecture after the closure of FTMH may involve the formation of a glial tissue which seals the hole at the outer layers. The FTMH shown in Fig. 6a closed within 2 months after surgery (3 months after the first examination). The closure was associated with a resolution of the cystic cavities between the OPL and HFL, and within the INL; this allowed a drop of the elevated inner layers of the foveal walls and a reattachment of the outer retina at the RPE. However, the central fovea remained elevated and did not contain an ONL and photoreceptors, but was filled by a tissue with medium reflectivity, likely composed of Müller cells. The ELM below this tissue was hyperreflective. Between 3 and 5 months, the gap in the central ONL closed, likely by a centripetal displacement of photoreceptor cell somata. This was associated with a drop of the central fovea and a decrease in the size of the photoreceptor-free area. During the next month, the foveal morphology did not alter, with the exception of a disappearance of the hyperreflectivity of the central ELM. There remained a small photoreceptor-free area in the foveal center, as indicated by the defect of the EZ line. The BCVA of this eye improved from 0.25 before to 1.0 after surgery.

The FTMH shown in Fig. 6b closed within 1.2 months after surgery (2.7 months after the first examination). The resolution of the cystic cavities in the foveal walls was associated with a reattachment of the central outer retina at the RPE. The central fovea did not contain an ONL and photoreceptors. The ELM, which spanned the photoreceptor-free area, was hyperreflective. The ONL-free foveal center was filled by a hyperreflective glial tissue. Between 2.7 and 14.5 months, the foveal shape regenerated; the gap in the central ONL closed which was associated with a thickening of the foveal center, a disappearance of the photoreceptor-free area, and a regeneration of the fovea externa which is visible at the inclined courses of the ELM and EZ lines in the foveola. The BCVA of this eye improved from 0.3 before to 0.6 after surgery.

Figure 6c shows another case of a foveal regeneration after surgical closure of a FTMH; vitrectomy with ILM peeling was performed 0.5 month after the first visit. The resolution of the cystic cavities in the foveal walls was associated with a drop of the elevated inner layers of the foveal walls and a reattachment of the outer retina at the RPE (2 months). There was a relatively large gap in the central ONL (diameter, 300 µm) which was photoreceptor-free. The gap was filled by a tissue of medium reflectivity, likely composed of Müller cells. Between 2 and 31 months after the first examination, the gap in the central ONL closed, likely by a centripetal displacement of photoreceptor cell somata. The BCVA of this eye improved from 0.25 before surgery to 0.5 at the end of the examination period.

## Discussion

**Fig. 7:**
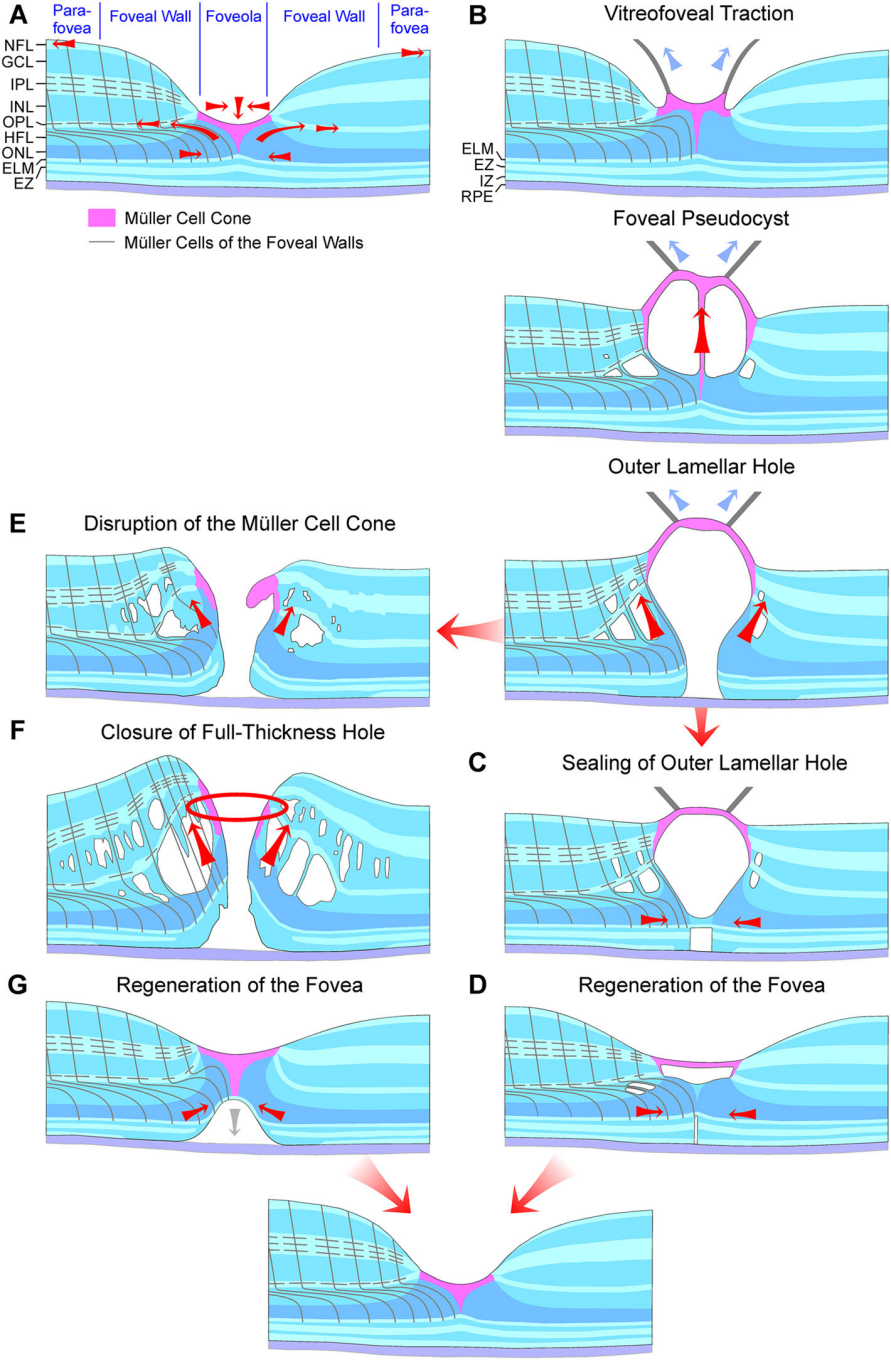
Hypothetical mechanisms of the formation and regeneration of outer lamellar and full-thickness macular holes. **a** Normal fovea. The Müller cell cone in the foveola is shown in pink. The black lines indicate the main and side processes of the *z*-shaped Müller cells of the foveal walls and parafovea. The arrows indicate traction vectors which are effective in the normal fovea. The fovea externa is formed by the elongated photoreceptor outer segments and is visible by the inclined courses of the external limiting membrane (ELM) and ellipsoid zone (EZ) lines. The horizontal layer of the Müller cell cone keeps the inner layers of the foveal walls together, and the vertical stalk of the Müller cell cone prevents an elevation of the inner layers of the foveal walls. The outer processes of the Müller cells of the foveal walls provide the structural stability of the outer layers, by tight-like junctions between these processes and photoreceptor cells, especially in the outer part of the outer nuclear layer (ONL), and by constitution of the ELM. Müller cells of the foveal walls may also regulate the slope of the ELM in the fovea externa. The side processes of the cells in the outer plexiform layer (OPL) are connected; horizontal contraction of these processes produces a centrifugal displacement of Henle fibers resulting in a steeper slope of the ELM in the fovea externa. **b**–**d** Formation (**b**) and closure (**c**) of outer lamellar holes, and regeneration of the foveal shape (**d**). The presumed Müller cell-mediated tissue movements are indicated by *red arrows*. *Blue arrows* indicate traction exerted by the posterior hyaloid. **e**–**g** Formation (**e**) and closure (**f**) of full-thickness macular holes, and regeneration of the foveal shape (**g**). GCL, ganglion cell layer; HFL, Henle fiber layer; INL, inner nuclear layer; IPL, inner plexiform layer; IZ, interdigitation zone; NFL, nerve fiber layer; RPE, retinal pigment epithelium

In the normal fovea, the Müller cell cone provides the resistance of the foveal tissue against mechanical stress resulting from anteroposterior and tangential tractions (Fig. 7a). It was shown that there is a lifelong slow increase in the area of the foveal avascular zone associated with a decrease in the thickness of the foveal walls [29]. This suggests that there is a continuous centrifugal traction which widens the foveal pit. The traction is likely exerted by nerve fibers and astrocytes and is counteracted by the horizontal layer of the Müller cell cone which keeps the inner layers of the foveal walls together. The stiffness of the inner plexiform layer [4] may support an elevation of the inner layers of the foveal walls. This is prevented by the vertical stalk of the Müller cell cone in the center of the foveola. Anteroposterior or tangential tractions which produce a detachment and elevation of the horizontal layer of the Müller cell cone, associated with an elongation or disruption of the stalk of the Müller cell cone, may also produce an elevation of the inner layers of the foveal walls which is often associated with a schistic tissue disruption between the OPL and HFL (Figs. 1h, 3j, 4a–c, 7b).

The present data may suggest that the Müller cell cone provides the foveal stability in cases of a cystic disruption of the foveola. In cases of a central photoreceptor degeneration without vitreomacular traction which may detach or tear the Müller cell cone as in macular telangiectasia type 2, the foveal contour remains normal (Fig. 1a–f). In cases of vitreomacular traction which detach the horizontal layer of the Müller cell cone from the HFL/ONL in the foveola, the foveal contour is altered (Fig. 1g–k). The detachment of the Müller cell cone from the HFL/ONL was explained by the fact that there are no cellular connections between the cells of the Müller cell cone and the outer processes of the Müller cells of the foveal walls which envelop the somata and fibers of the photoreceptor cells in the HFL/ONL [4, 13]. The outer layers of the foveola (HFL, ONL) were suggested to be structurally stabilized by the outer processes of the Müller cells of the foveal walls and parafovea, via a centripetal traction at the ELM and the outer part of the ONL and a centrifugal traction on the Henle fibers resulting from a horizontal contraction of Müller cell side processes in the OPL (Fig. 7a) [4]. The finding that, after a tractional disruption of the Müller cell cone, the shape of the ONL in the foveola may remain intact (with the exception of a widening of the central ONL; Fig. 3b, d–f) could be explained with the fact that the ONL is not stabilized by the Müller cell cone, but by the outer processes of the Müller cells of the foveal walls which also constitute (together with photoreceptor cells) the ELM (Fig. 2). The data may suggest that the horizontal layer of the Müller cell cone also counteracts the centrifugal pulling of the Henle fibers produced by the Müller cells of the foveal walls which may mediate the widening of the central ONL.

Macular telangiectasia type 2 was suggested to be caused by a dysfunction and/or degeneration of Müller cells; Müller cell dysfunction may produce the vascular symptoms and the degeneration of photoreceptors [30, 31]. The finding that the foveal shape is maintained by the Müller cell cone in cases of macular telangiectasia type 2 (Fig. 1a–f) suggests that the loss of photoreceptor cells is associated with a degeneration of the outer processes of Müller cells of the foveal walls which surround the photoreceptor cells in the central outer retina [4, 13] but not by a dysfunction/degeneration of the cells of the Müller cell cone. This assumption also fits with the facts that Müller cells of the foveal walls and parafovea, but not cells of the Müller cell cone (which lies within the foveal avascular zone), have contact to blood vessels [13] and that the Müller cell markers vimentin and glutamine synthetase are reduced in the fovea of a macular telangiectasia type 2 patient compared to control tissues while the expression of glial fibrillary acidic protein (GFAP) is normal in the macular region [30, 31]. Glutamine synthetase is normally expressed by the Müller cells of the foveal walls and parafovea but at low level in the Müller cell cone (because the Müller cell cone has no contact to retinal synapses), whereas GFAP is expressed by the Müller cell cone and, at lower level, by the outer processes of the Müller cells of the foveal walls [4, 32, 33]. In addition, the previously published finding that markers for the ELM are lost in the central macula of the macular telangiectasia type 2 patient [31] suggests a dysfunction and/or degeneration of the Müller cells of the foveal walls and parafovea because cells of the Müller cell cone likely do not contribute to the formation of the ELM because they have no direct contact to the photoreceptor cells [13].

In various types of foveal defects, which are characterized by a detachment and/or a disruption of the Müller cell cone, Müller cells of the foveal walls may support the structural stability of the foveal center (Fig. 3b, d–m). These cells may also seal holes in the central outer retina by the formation of a tissue band which bridges the holes at the ELM (Fig. 4a, c, d) and may mediate the regeneration of the central fovea in cases of outer and tractional lamellar holes (Figs. 4b, e, 5a). It was shown that the regeneration of the foveal structure after surgical or spontaneous closure of FTMH may include a centripetal displacement of the central ONL, likely caused by radial forces created by the contraction of Müller cell processes in the outer part of the ONL and at the ELM which drag the photoreceptor cell somata centrally [18, 19]. The Müller cell-mediated centripetal displacement of the photoreceptor cell somata results in a closure of the central photoreceptor-free area and an increase in the photoreceptor density in the regenerated foveola. A similar centripetal displacement of the central ONL was found in the cases of the regeneration of the foveal structure after surgical closure of FTMH described in this study (Fig. 6a–c). In the case of the tractional lamellar hole shown Fig. 4b, the hole in the central outer retina was likely closed by a centripetal displacement of the photoreceptor cell somata. In the cases of outer lamellar holes (Fig. 4a, c, d) and the regeneration of a serous macular detachment (Fig. 5a), the diameter of the hole in the outer retina decreased time dependently by a centripetal displacement of the ONL.

A tractional disruption of the Müller cell cone is often associated with a dehiscence of the central ONL; the foveal center is only stabilized by the ELM (Fig. 3c–f). The regeneration of the foveal center proceeds by a centripetal Müller cell-mediated displacement of photoreceptor cell somata which may occur despite the disruption of the Müller cell cone, as shown in Fig. 3f. It was shown that the closure of a FTMH can occur although the inner Müller cell layer of the foveola remains detached from the central ONL; in this mode of hole closure, the ONL is displaced centripetally, the hole closes only at the ELM, and a new Müller cell cone is not formed [9]. Furthermore, a regeneration of the central ELM and the foveal shape can also proceed without a centripetal displacement of the ONL, as shown in Fig. 4f.

Shortly after the closure of a FTMH, the central ONL displayed a gap and an absence of photorecpetors while the gap in the ELM was closed (Fig. 6a–c). The gap in the central ONL was filled by a tissue of medium reflectivity, likely Müller cells. On the other hand, in cases of outer lamellar holes, the holes in the outer retina were sealed by a hyperreflective tissue band at the ELM and the outer part of the ONL (Fig. 4a–d). Similar hyperreflective bands at the ELM, which sealed a hole in the central ONL, were found in different types of outer macular defects (Fig. 3a, d–j, m) and in the case of the foveal regeneration after serous macular detachment (Fig. 5a). It is likely that the tissue bands provide the structural stability of the outer retina when a hole in the central ONL is present. As suggested by the cases shown in Figs. 4b and 5a, the tissue bands at the ELM may be also implicated in the regeneration of the regular structure of the outer retina; a contraction of the bands may support the centripetal displacement of the ONL which closes the central photoreceptor-free area. However, a new ELM can be also formed without a displacement of the ONL, as indicated by the case of an outer lamellar hole shown in Fig. 4f.

The cellular origin of the hyperreflective tissue bands at the ELM is unclear. The central fovea only contains Müller and photoreceptor cells [1, 4, 13]. Because the holes in the central ONL were associated with an absence of photoreceptors, as indicated by the defects of the EZ and IZ lines, the hyperreflective tissue bands which bridge the holes are formed by Müller cells. The tissue bands are formed at the outer part of the ONL and the ELM. The ELM is constituted by tight-like junctions between Müller cell processes and photoreceptor cells [14, 15]. Because the fibers and somata of the photoreceptor cells are surrounded by the outer processes of the Müller cells of the foveal walls [4, 13], it is likely that the hyperreflective tissue bands in the outer retina are formed by outgrowing processes of these cells. The hyperreflectivity of the bands is likely generated by light reflections at the outgrowing Müller cell processes which are optically dense due to an upregulation of glial intermediate filaments. Müller cell gliosis is known to be associated with an upregulation of intermediate filaments [34]; these filaments support the structural stabilization of the retina [3, 35–37].

In most cases described in this study, the formation of glial tissue bands which seal outer retinal holes at the ELM was associated with a narrowing of the hole by the Müller cell-mediated centripetal displacement of the ONL. It was shown that small FTMH with a diameter of less than 400 µm may close spontaneously [38, 39]. The finding that only small holes can close spontaneously was explained with the assumption that the distance of the Müller cell-mediated centripetal tissue movements is restricted to the diameter of the foveola (in the mean, about 350 µm [40]) [18]. It is unclear whether the size of an outer lamellar hole plays a role in the sealing of the holes by a glial tissue band at the ELM. In the cases of outer lamellar holes presented in this study, the diameters of the holes ranged from 75 to 290 µm before the formation of the tissue bands and from 40 to 200 µm after the formation.

Figure 7b–g summarizes hypothetical mechanisms of the formation and closure of outer lamellar holes and FTMH. The tractional formation of a foveal pseudocyst results in a detachment and elevation of the inner Müller cell layer of the foveola associated with an elongation and subsequent disruption of the stalk of the Müller cell cone (Fig. 7b) [18]. The elevation of the inner layer of the foveola produces an elevation of the inner layers of the foveal walls which is associated with a cystic disruption of the tissue mainly between the OPL and HFL. The position of the Henle fiber bundles, which traverse the cystic cavities, may indicate that the Müller cells of the foveal walls are stretched which causes a shape alteration of the Müller cells from the z-shape to a straight shape [11]. The stretched Müller cells were suggested to exert a traction onto the outer retina in an oblique anterior direction which causes a dehiscence of the central ONL [11] resulting in the formation of an outer lamellar hole (Fig. 7b). The hole is closed by two mechanisms: a centripetal displacement of the photoreceptor cell somata in the ONL mediated by a concentric contraction of Müller cell processes near and at the ELM, and the formation of a tissue band at the ELM by outgrowing Müller cell processes which bridges the hole (Fig. 7c, d). Contraction of the glial tissue bands may support the centripetal displacement of the photoreceptor cell somata. Tractional disruption of the Müller cell cone produces a FTMH from an outer lamellar hole (Fig. 7e). The hole is widened by the enlargement of edematous cysts in the foveal walls which further elevates the inner layers of the walls (Fig. 7f) [11, 41, 42]. The closure of FTMH was suggested to proceed by a concentric contraction of Müller cell side processes in the OPL (Fig. 7f) and the resolution of the edematous cysts in the foveal walls; the subsequent regeneration of the foveal shape proceeds via a Müller cell-mediated centripetal displacement of the photoreceptor cell somata in the ONL (Fig. 7g) [18].

In summary, the present data suggest that the Müller cell cone maintains the structure of the central fovea in cases of a cystic disruption of the foveola when no tractions are present which detach or disrupt the cone. Müller cells of the foveal walls may provide the stability and may mediate the regeneration of the outer fovea in cases of defects of the central ONL by two mechanisms: a centripetal displacement of the photoreceptor cell somata and the formation of a glial tissue band at the ELM and the outer part of the ONL which bridges the hole in the central ONL.

## Abbreviations

BCVA: Best-corrected visual acuity
ELM: External limiting membrane
EZ: Ellipsoid zone
FTMH: Full-thickness macular hole
HFL: Henle fiber layer
ILM: Internal limiting membrane
INL: Inner nuclear layer
IZ: Interdigitation zone
LHEP: Lamellar hole-associated epiretinal proliferation
NFL: Nerve fiber layer
ONL: Outer nuclear layer
OPL: Outer plexiform layer
RPE: Retinal pigment epithelium
SD-OCT: Spectral-domain optical coherence tomography
